# Evolution of H5-Type Avian Influenza A Virus Towards Mammalian Tropism in Egypt, 2014 to 2015

**DOI:** 10.3390/pathogens8040224

**Published:** 2019-11-07

**Authors:** Sara Hussein Mahmoud, Ahmed Mostafa, Rabeh El-Shesheny, Mohamed Zakaraia Seddik, Galal Khalafalla, Mahmoud Shehata, Ahmed Kandeil, Stephan Pleschka, Ghazi Kayali, Richard Webby, Veljko Veljkovic, Mohamed Ahmed Ali

**Affiliations:** 1 Center of Scientific Excellence for Influenza Viruses, National Research Centre (NRC), Dokki, Giza 12622, Egypt; sarahussein9@yahoo.com (S.H.M.); ahmed_elsayed@daad-alumni.de (A.M.); ra_eny@yahoo.com (R.E.-S.); shehata_mmm@hotmail.com (M.S.); kandeil_a@hotmail.com (A.K.); 2 Institute of Medical Virology, Justus Liebig University (JLU) Giessen, Schubertstrasse 81, 35392 Giessen, Germany; stephan.Pleschka@viro.med.uni-giessen.de; 3 Department of Infectious Diseases, St. Jude Children’s Research Hospital, Memphis, TN 38105, USA; richard.webby@stjude.org; 4 Microbiology Department, Faculty of Agriculture, Cairo University, Giza 12613, Egypt; mzsedik@yahoo.com (M.Z.S.); galal1950@hotmail.com (G.K.); 5 Department of Epidemiology, Human Genetics, and Environmental Sciences, University of Texas, Houston, TX 77030, USA; ghazi@human-link.org; 6 Human Link, Hazmieh 1109, Lebanon; 7 Biomed Protection, Galveston, TX 77550, USA

**Keywords:** ISM analysis, evolution, human-type receptor

## Abstract

Highly pathogenic avian influenza viruses (HPAIV) of the H5-subtype have circulated continuously in Egypt since 2006, resulting in numerous poultry outbreaks and considerable sporadic human infections. The extensive circulation and wide spread of these viruses in domestic poultry have resulted in various evolutionary changes with a dramatic impact on viral transmission ability to contact mammals including humans. The transmitted viruses are either (1) adapted well enough in their avian hosts to readily infect mammals, or (2) adapted in the new mammalian hosts to improve their fitness. In both cases, avian influenza viruses (AIVs) acquire various host-specific adaptations. These adaptive variations are not all well-known or thoroughly characterized. In this study, a phylogenetic algorithm based on the informational spectrum method, designated hereafter as ISM, was applied to analyze the affinity of H5-type HA proteins of Egyptian AIV isolates (2006–2015) towards human-type cell receptors. To characterize AIV H5-HA proteins displaying high ISM values reflecting an increased tendency of the HA towards human-type receptors, recombinant IV expressing monobasic, low pathogenic (LP) H5-HA versions in the background of the human influenza virus A/PR/8/1934(H1N1) (LP 7+1), were generated. These viruses were compared with a LP 7+1 expressing a monobasic H5-HA from a human origin virus isolate (human LP-7271), for their receptor binding specificity (ISM), in vitro replication efficiency and in vivo pathogenicity in mammals. Interestingly, using ISM analysis, we identified a LP 7+1 virus (LP-S10739C) expressing the monobasic H5-HA of AIV A/Chicken/Egypt/S10739C/2015(H5N1) that showed high affinity towards human-type receptors. This in silico prediction was reflected by a higher in vitro replication efficiency in mammalian cell cultures and a higher virulence in mice as compared with LP-7271. Sequence comparison between the LP-S10739C and the LP-7271 H5-HA, revealed distinct amino acid changes. Their contribution to the increased mammalian receptor propensity of LP-S10739C demands further investigation to better deduce the molecular determinant behind the reported high morbidity of 2014 to 2015 HPAI H5N1 virus in humans in Egypt. This study provides insights into the evolution of Egyptian H5 HPAIVs and highlights the need to identify the viral evolution in order to recognize emerging AIV with the potential to threaten human and animal populations.

## 1. Introduction

Avian influenza viruses (AIVs) represent a continual challenge to human and animal health worldwide [1]. The segmented genome and the lack of proof-reading activity of influenza viral polymerase enable AIVs to evolve genetically in their natural reservoir (e.g., aquatic waterfowl) or domestic poultry via exchange of their viral genome segments or by acquisition of adaptive amino acid (aa) changes. This alteration can alter viral host tropism and transmissibility of the nascent reassortant or variant AIVs to other hosts including humans. Additionally, the genetic variations in the genome of AIVs allows the virus to escape the pressures imposed by the pre-existing neutralizing antibodies (immunity) or antiviral drugs [1].

Historically, pandemic influenza viruses arose and turned from natural and avian reservoirs to the human population [2]. The major genetic changes, acquired by pandemic strains, were mainly localized to the viral hemagglutinin (HA) protein. The HA is a surface glycoprotein that mediates binding of IAV to the cellular receptor [3]. Specific substitutions in the aa sequences of HA can allow them to switch from their target receptors from an avian-type receptor [α2,3-linked sialic acid (α2, 3-SA)] to a human-type receptor [α2,6 linked sialic acid (α2,6-SA)], generating new virus variants with efficient poultry-to-human transmissibility [4,5,6]. The recognition of a human-type receptor by the HA of AIVs strongly enhances the zoonotic potential of these viruses, giving rise to the evolution of candidate pandemic strains (CPS). Timely prediction of these CPS, against which there is no pre-existing protective immunity, poses a great challenge that can drastically affect human populations due to the time gap between the molecular identification and genetic characterization of a new IAV strain and the production of a relevant effective vaccine.

For a decade, Egypt has reported the highest morbidity and mortality rates following infection with the H5N1-type highly pathogenic avian influenza A viruses (H5N1-HPAIV) worldwide. Phylogenic analysis of H5N1-HPAIV in Egypt revealed their evolution since 2006 (clade 2.2) into two new subclades (2.2.1, 2.2.1.1, and 2.2.1.2). Viruses of subclades 2.2.1.1 and 2.2.1.2 showed altered virulence, pathogenicity, transmissibility, receptor-binding specificity, and antiviral drug sensitivity [7,8]. During 2014 to 2015 alone, the WHO recorded approximately 173 cases and 53 deaths in Egypt [9]. To this point, there is an urgent need for an early alert system to predict the pandemic potential of the 2014 to 2015 AIVs in the presence of representatives for the previous era and clades (2006 to 2013).

For developing countries, bioinformatics represents an economical tool as a “flu alert” to estimate the zoonotic potential of AIVs towards mammals including humans and to identify their pandemic risk. In this study, we used a specific bioinformatics algorithm to determine the evolution of AIVs in their hosts toward mammals. This novel phylogenetic algorithm is based on the informational spectrum method (ISM) and was employed to describe the receptor binding characteristics of AIVs, isolated in Egypt from 2006 to 2015 representing different genetic clades with varying affinity towards mammals [10,11,12,13].

## 2. Results

### 2.1. ISM Analysis and Generation of Selected Reassortant Strains

Previously, it has been shown that the interactions between the hemagglutinin (HA) from H5N1 influenza viruses and avian and human receptors were characterized by the specific ISM frequencies F (0.076) and F (0.236), respectively [10]. It has also been shown that mutations, which increase the ratio of the amplitudes of these frequencies, AF (0.236)/AF (0.076), significantly increase the human propensity of H5N1 viruses [13]. 

**Figure 1:**
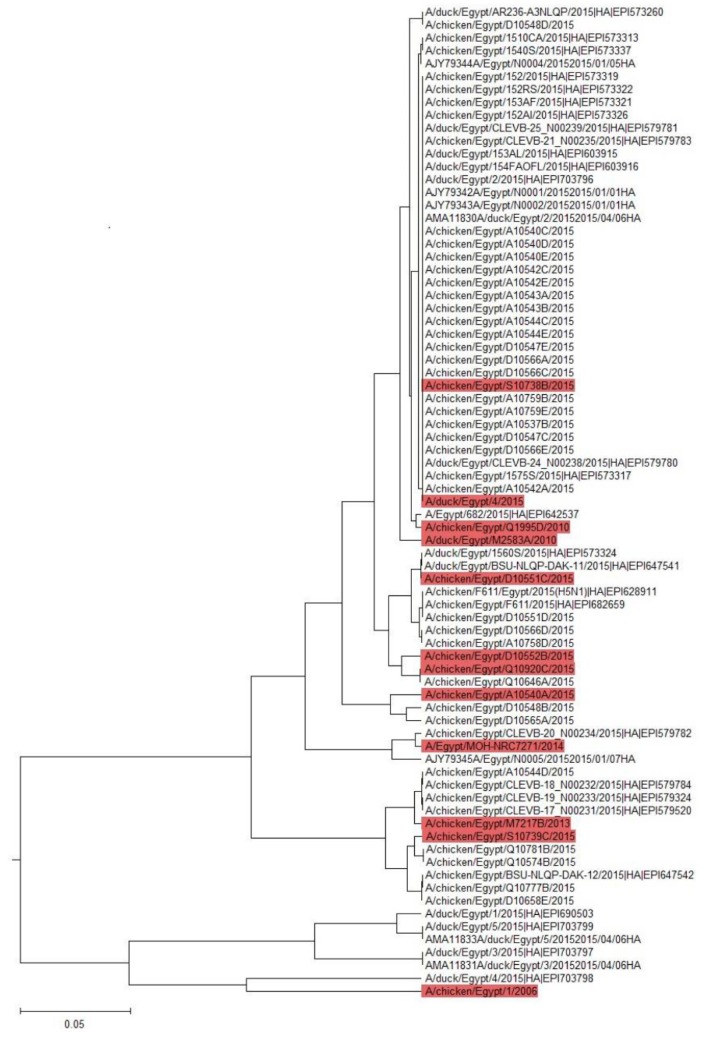
ISM phylogenetic analysis of the avian 2014 to 2015 H5-HA viruses´ affinity to mammals. On the basis of the ISM analysis, seven viruses were selected from different clusters to be tested in vitro, (S10738B, D10551C, D10552B, Q10920C, A10540A, S10739C, and A/du/Eg/4/2015).

We generated an ISM-based phylogenetic tree for the HA of H5N1 viruses collected in Egypt between 2014 and 2015, using the amplitude ratio, AF (0.236)/AF (0.076), as the distance measure. The results obtained by ISM analysis of the HA amplitude ratio of different AIVs showed variable affinity clusters of HA proteins from 2014 to 2015 towards mammalian cell receptors. Therefore, a representative H5N1 virus (clade 2.2.1.2) from each cluster was selected for further experimental validations (A/chicken/Egypt/A10540A/2014, A/chicken/Egypt/D10551C/2014, A/chicken/Egypt/D10552B/2015, A/chicken/Egypt/S10738B/2015, A/chicken/Egypt/S10739C/2015, A/chicken/Egypt/Q10920C/2015, and A/duck/Egypt/4/2015) (Figure 1).

The following viruses were compared with other strains isolated from 2006 to 2013 for different clades as controls: influenza A/chicken/Egypt/1/2006 (clade 2.2.1), A/chicken/Egypt/Q1995D/2010 (clade 2.2.1.1), A/duck/Egypt/M2583A/2010 (clade 2.2.1.1), and A/chicken/Egypt/M7217B/2013 (clade 2.2.1.2)), in addition to the human strain A/Egypt/MOH-NRC7271/2014 (clade 2.2.1.2) (Table 1). To experimentally validate the variable impact of selected HA H5 proteins on their affinity towards mammals by in vitro experiments, we first generated recombinant low pathogenic H5N1 AIVs of selected strains (2006 to 2015) expressing H5-HA proteins with a modified and monobasic cleavage site as a safety measure and the other viral proteins from influenza A/PR/8/1934 (H1N1) segments to generate low pathogenic reassortants (LP 7+1). A LP 7+1, expressing a monobasic H5-HA (human LP-7271) of human origin, was also generated. Titers of the different virus stocks were determined on MDCK-II cells and varied between 10^6.4^ TCID_50_/100 µL and 10^2.42^ TCID_50_/100 µL (Table 1). 

### 2.2. Receptor-Binding Specificity for LP (7+1) H5 Reasstortants

**Figure 2:**
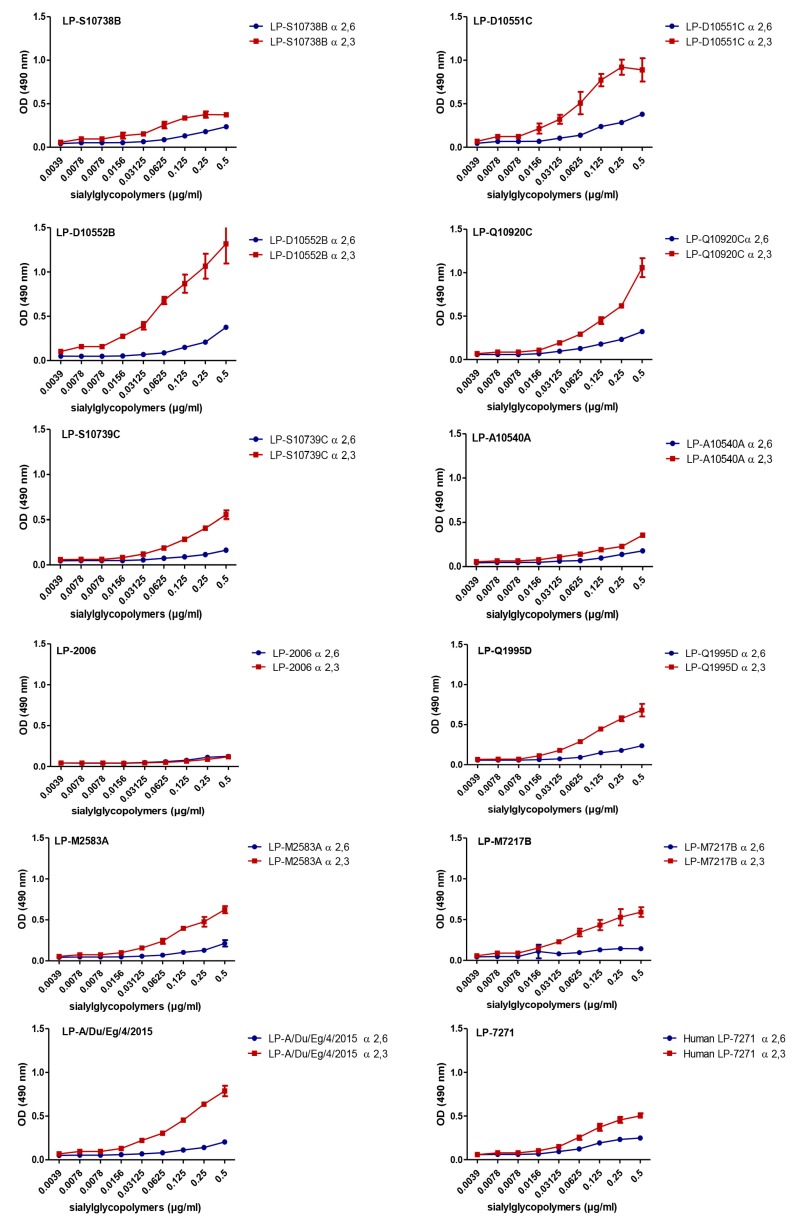
Receptor binding specificity of avian H5-HA viruses to mammalian- and avian-type receptors. The PR8 viruses expressing monobasic HA from clade 2.2.1.2 H5N1 strains (LP-S10738B, LP-D10551C, LP-D10552B, LP-Q10920C, LP-S10739C, LP-A10540A, and LP-A/Du/Eg/4/2015); clade 2.2.1 (LP-2006), and clade 2.2.1.1 (LP-Q1995D, LP-M2583A, and LP-M7217B), were compared to LP-7271 (clade 2.2.1.2, H5 HA of human origin) using solid-phase receptor binding assay.

To experimentally assess the differential tendency of the H5-HA proteins towards mammalian cells (Figure 1), we analyzed the affinity of the LP 7+1-PR8 reassortants expressing the different predefined monobasic H5-HA proteins to avian (α2,3-SA) and human (α2,6-SA) type receptors. Unexpectedly, in the solid phase binding reactions, there are no obvious strains that favor α2,6-SA, as related to that of human influenza A/Egypt/MOH-NRC7271/2014 (H5N1, human LP-7271, clade 2.2.1.2). The data obtained revealed that the H5-HA proteins, derived from influenza A/chicken/Egypt/D10551C/2014 (H5N1, LP-D10551C, clade 2.2.1.2), A/chicken/Egypt/D10552B/2015 (H5N1, LP-D10552B, clade 2.2.1.2), A/chicken/Egypt/Q10920C/2015 (H5N1, LP-Q10920C, clade 2.2.1.2), and A/duck/Egypt/4/2015 (H5N1, LP-A/Du/Eg/4/2015, clade 2.2.1.2) of clade 2.2.1.2, demonstrated comparable to slightly lower affinities for human-type receptors but higher affinities to avian-type receptors, as compared with those for human LP-7271, respectively (Figure 2). However, the affinities of the H5-HA proteins of influenza A/chicken/Egypt/S10738B/2015 (H5N1, LP- S10738B, clade 2.2.1.2), A/chicken/Egypt/A10540A/2014 (H5N1, LP-A10540A, clade 2.2.1.2), and A/chicken/Egypt/S10739C/2015 (H5N1, LP-S10739C, clade 2.2.1.2) to avian- and human-type receptors were slightly lower to comparable, respectively, as compared with the affinity of the human LP-7271 for both receptors (Figure 2). 

Except for the H5-HA of influenza A/chicken/Egypt/1/2006 (H5N1, LP-2006, clade 2.2.1), which showed a lower affinity to human- and avian-type receptors than human LP-7271, the H5-HA proteins from influenza A/chicken/Egypt/Q1995D/2010 (H5N1, LP-Q1995D, clade 2.2.1.1), A/duck/Egypt/M2583A/2010 (H5N1, LP-M2583A, clade 2.2.1.1), and A/chicken/Egypt/M7217B/2013 (H5N1, LP-M7217B, clade 2.2.1.2) demonstrated binding affinity equal to that of human LP-7271 and were able to bind both types of receptors (Figure 2).

### 2.3. Replication Efficiency of LP 7+1 Reassortants In Vitro

**Figure 3:**
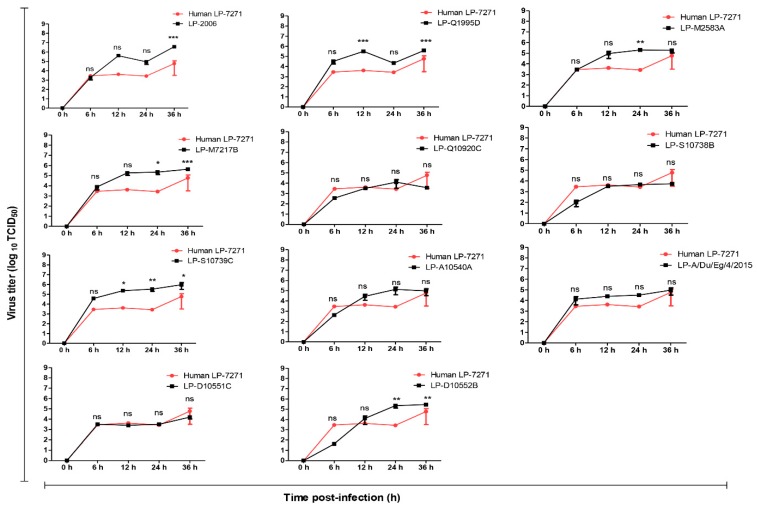
Replication kinetics of LP H5-HA viruses in MDCK-SIAT1 cells. The cells were infected with the tested viruses at different time points (6, 12, 24, and 36 h) at a MOI of 0.001. Unlike other PR8 expressing the 2014 to 2015 H5 HA, the LP-S10739C replicated significantly higher than LP-7271 at different time points 12, 24, and 36 h p.i. Error bars reflect standard deviation (SD) of three independent experiments. Statistical analysis was performed using repeated measures ANOVA, followed by Bonferroni post-hoc test. The significant differences are indicated (* = *p* < 0.05, ** = *p* < 0.01, *** = *p* < 0.001, and nonsignificant = ns).

The replication of LP 7+1 reassortant viruses carrying different H5-HAs was investigated further in two in vitro mammalian cell-culture systems (i) MDCK-SIAT1 cells, over-expressing α2,6-SA [14,15], and (ii) A549 cells, expressing similar amounts of α2,3-SA and α2,6-SA [13]. In comparison to the human LP-7271 (Figure 3), LP 2006, LP-Q1995D, LP-M7217B, 2014 to 2015 LP-S10739C, and LP-D10552B replicated to significantly higher titers in MDCK-SIAT1 cells at 12 to 36 h and 24 to 36 h post-infection, respectively. The replication efficiencies of LP 7+1 viruses expressing monobasic H5-HA of 2014 to 2015 viruses were comparable to human LP-7271, but lower than 2006 (LP-2006), 2010 (LP-Q1995D), and 2013 (LP-M7217B) at 6 to 36 h p.i. (Figure 3). 

**Figure 4:**
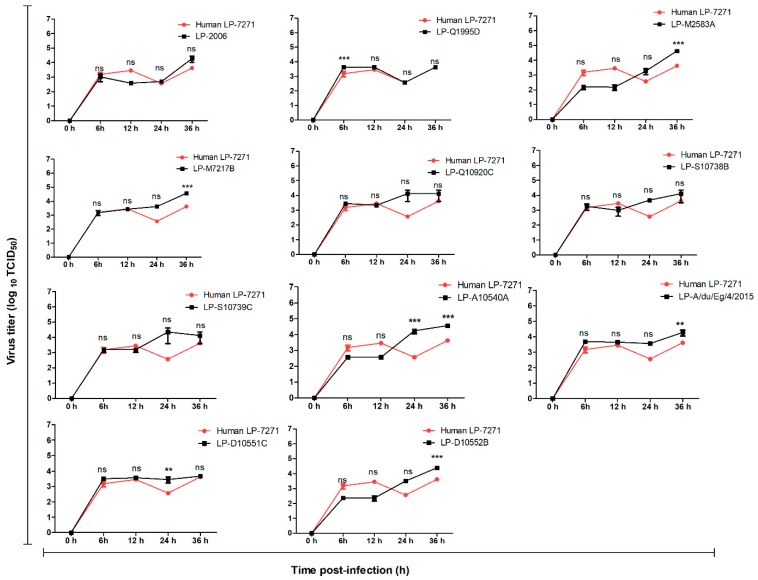
Replication kinetics of LP H5-HA viruses in A549 cells. The cells were infected with the tested viruses at different time points (6, 12, 24, and 36 h) at a MOI of 0.001. All 2014 to 2015 viruses were highly replicating than human LP-7271 strain especially at 24 h p.i. the replication efficiencies of LP-A10540A, LP-D10551C, LP-D10552B, and LP-A/du/Eg/4/2015 were significantly higher than LP-7271 at 24 h or 36 h p.i. Error bars reflect standard deviation (SD) of three independent experiments. Statistical analysis was performed using repeated measures ANOVA, followed by Bonferroni post-hoc test. The significant differences are indicated (* = *p* < 0.05, ** = *p* < 0.01, *** = *p* < 0.001, and nonsignificant = ns).

Interestingly, in human A549 cells, all of the LP 7+1 virus replicated to comparable, or slightly higher, titers than the human LP-7271 at early time points (6 to 12 h) (Figure 4). At later time points (24 to 36 h), LP-M7217B, LP-M2583A, LP-D10552B, LP-D10551C, LP-A/Du/Eg/4/2015, LP-S10739C, and LP-A10540A showed significantly improved replication kinetics as compared with the control LP-7271.

### 2.4. Pathogenicity of LP 7+1 H5-Reasstortants In Vivo

**Figure 5:**
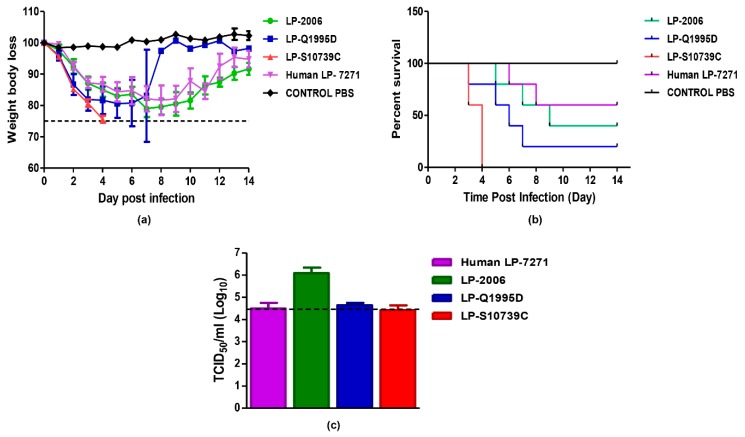
Virulence of LP AIVs in mice. Each mouse was infected with 30 µL of 104.25 TCID_50_ /100 µL. The morbidity (weight body loss) (**a**) and mortality (percent survival) (**b**) rates were monitored within 14 days. (**c**) virus shedding in mice lungs. Mice were sacrificed three days p.i and lungs were preserved in DMEM and viral loads were detected using TCID_50_ assay.

To assess the in vivo pathogenicity of LP 7+1 H5-HA viruses, five groups of C57BL/6 female mice were infected with selected LP 7+1 H5-HA expressing reassortants of 2006 (LP-2006), Q1995D (LP-Q1995D), S10739C (LP-S10739C), and MOH-7271 (human LP-7271, positive control). In parallel, one group was inoculated with sterile 1X PBS as a negative control. The LP-S10739C virus showed the highest morbidity (Figure 5a) and mortality (Figure 5b) in mice as compared with human LP-7271. All mice, infected with LP-S10739C, died naturally after the fourth day (Figure 5). However, in the second group, inoculated with LP-Q1995D, mice naturally died or had to be euthanized (weight body loss ≥ 25%). Viral titers from lung homogenates of infected mice at 3 dpi were also quantified using TCID_50_ assay. Although LP-S10739C showed the highest virulence, the infection was accompanied with the lowest virus shedding in the lung of infected mice (Figure 5c).

## 3. Discussion

Since 1997, HPAIV H5N1 viruses started to vanquish species barriers and transmitted to humans, leading to the highest mortality rates caused by zoonotic AIV in humans. Since 2006, Egypt has been the country most strongly impacted by human infections with HPAIV H5N1 recording the highest morbidity and mortality rates (Egyptian cases = 359 (860 cases worldwide), Egyptian deaths = 120 (454 deaths worldwide), and case fatality rate = 53.2) [16]. This zoonotic potential of these viruses is based on their specific genetic determinants, which have been studied over the last two decades. Most of the current studies, which discuss the evolution of HPAIV H5N1 viruses towards mammals, are carried out with human H5N1-type isolates, which may possess additional host-specific adaptation characteristics despite their H5-type HA [13,17,18]. Therefore, there is a strong demand to develop new and economic bioinformatics-based tools to allow early recognition of the zoonotic risk potential of HPAIV H5N1 strains that might be a pacemaker for an IAV pandemic. To this point, various in silico and phylogenetic studies have been undertaken to monitor the evolution of AIV isolates in avian species towards mammalian hosts. Nevertheless, only a few in silico studies have predicted the evolution of HPAIV H5N1 viruses in avian hosts towards mammals by altering the receptor specificity from avian receptors towards mammalian receptor specificity [10,13,19]. In this study, the ISM bioinformatics tool was used to detect changes in the LP H5-HA and their effect on the affinity towards human receptors that would likely have increased the pandemic potential of HPAIV strains between 2014 and 2015. Changes in replication efficiencies of AIV towards mammals was deduced by an increase of the amplitude ratio A (0.236)/(0.076) at the frequencies F at 0.236 and 0.076, the latter indicating critical interactions of the viral HA protein of IAV with human- and avian-type receptors, respectively [13]. During 2014 to 2015, Egypt experienced high morbidity rates due to AIV infections in humans [1,8,20]. HPAVI H5N1 isolated from 2006 to 2014 viruses acquired aa mutations that affected viral virulence, pathogenicity, transmission, and receptor-binding preference resulting in viruses that were genotypically diverged from previous strains [8].

The evolution of the HA subunit 1 (HA1) of Egyptian HPAIV H5N1 after 2009, revealed that the H5-HA acquired new informational spectrum (IS) properties that were predicted to significantly potentiate human tropism and pandemic risk [10]. All of the viruses tested displayed four characteristic mutations (D43N, S120D, S129Δ, and I151T) (Table 2), three of which were previously reported to increase binding to the human receptor [18,21]. This study also pointed out specific aa changes in the HA, which might contribute to the evolution of the 2014 to 2015 HPAIV H5N1 viruses towards replication potential in humans. However, previous studies rarely predicted changes, which could potentiate the interspecies transmission of these AIVs. Instead, such studies discussed the shift of HPAIV H5N1 from avian species to mammals by focusing mainly on the acquired aa mutations and variations following transmission to and adaptation in mammals (e.g., human isolates) [22]. In contrast, this study used a novel and economic bioinformatics tool, the ISM, to predict and identify variations in the HA of avian H5N1 isolates from the 2014 to 2015 season that had the ability to increase the human propensity of these virus strains. By applying the ISM to HPAIV H5N1 2014 to 2015 isolates, different genetic clusters of AIVs that differ in their affinities to mammalian cells were observed. Furthermore, different HA clusters (low, moderate, and high affinity for human-type receptors) represented by seven viruses were experimentally compared in their affinity either to avian-type (α2, 3-SA) or human-type (α2,6-SA) receptor. 

The relative binding affinities of the H5 viruses HA to α2,3-SA and to α2,6-SA, as related to that of human LP-7271, demonstrated that there are no obvious strains that favor α2,6-SA. However, the H5-HA proteins, derived from several tested strains including LP-D10551C, LP-Q10920C, LP-A/Du/Eg/4/2015, and LP-D10552B, have higher affinity to α2,3-SA. Interestingly, compared to LP-7271, LP-S10739C seemed to have slightly lower affinity for α2,6-SA, but comparable affinity for binding to α2,3-SA. LP-Q1995D, was highly similar in α2,3-SA and α2,6-SA binding to the human isolate LP-7271 as well. Eventually, LP-2006 was outperformed by LP-7271 in both ligands. Unfortunately, the control LP-7271, which assumingly has enough affinity for human ligand was not really improved upon additional mutations predicted by the algorithm based on the generic ligands used. Therefore, we demonstrated the in vitro interaction of the recombinant PR8 viruses expressing different clusters of H5 HA proteins using A549 and MDCK-SIAT1 cells. The A549 cells were previously shown to express human- and avian-type receptors at high levels [13], whereas MDCK-SIAT1 were engineered to overexpress human-type receptors [13,15].

The replication efficiency results showed that LP-S10739C, LP-A10540A, LP-D10552B, and LP-A/Du/Eg/4/2015 have a higher replication rate in MDCK-SIAT1 cells at 24 h p.i. than the human LP-7271 isolate. LP-S10739C replicated to higher titers at 12, 24, and 36 h p.i. as compared with the human LP-7271, while LP-D10552B showed higher titers only at 24 and 36 h p.i. Other viruses from different clades (LP-2006, LP-Q1995D, LP-M2583A, and LP-M7217B) showed higher replication rates than the human LP-7271 at early time points and comparable rates at later time points. In A549 cells, the majority of viruses expressing H5 HA from clade 2.2.1.2 (LP-Q10920C, LP-S10738B, LP-S10739C, and LP-A10540A) demonstrated a higher replication rates than the human LP-7271 isolate. This could confirm that the evolution of hemagglutinin (HA) protein of AIVs in avian hosts is probably enough to promote genetic and phenotypic changes, fulfilling the minimum zoonotic potential requirements that are needed for transmission and replication in mammalian systems without prior adaptation in a mammalian host [1].

In 2012, several reports demonstrated that only a limited number of adaptive genetic changes are sufficient to allow airborne transmission of HPAI H5N1 viruses and to acquire the competence to bind to human-type receptors [23]. In Egypt, at least two of the four (or five) mutations, needed to confer ferret-to-ferret airborne transmissibility, were reported in HPAI H5N1 isolates from poultry in Egyptian backyards and farms [13,23]. Accordingly, the H5-HA viruses of this study were genetically analyzed to detect variations in their aa sequences. In comparison to human LP-7271, the examination revealed aa substitutions in various positions (C4G, L297F, R373K, and S537F) of the LP-S10739C H5-HA protein (Table 2). To better assess the contribution of these (adaptive) changes in high risk potential infection scenarios, a thorough understanding for the implications of these four aa alterations on viral characteristics of HPAI H5N1 viruses seems to be of great importance.

**Figure 6:**
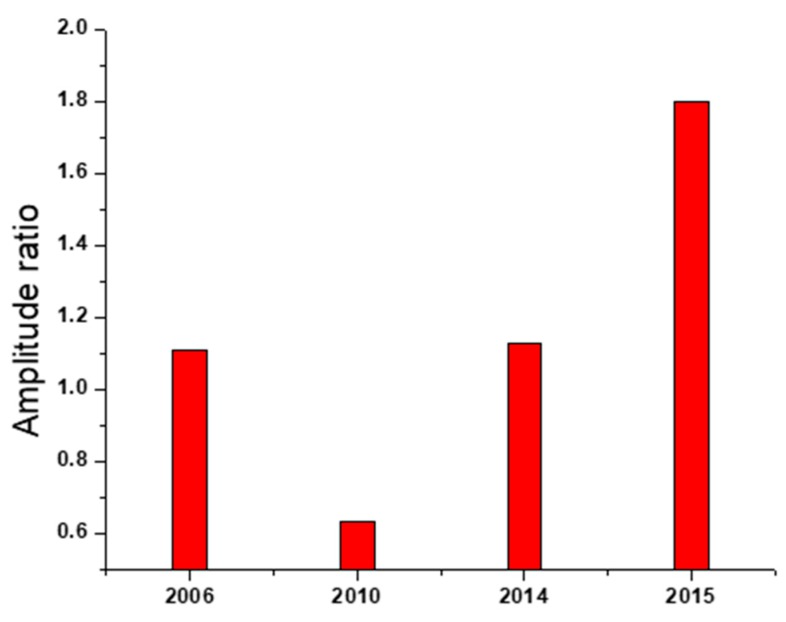
ISM analysis of avian HPAIV H5N1 viruses representing different clades emerged in Egypt since 2016. The data confirm the general higher tendency of the 2014 to 2015 HPAIV H5N1 isolated from Egypt towards mammalian-type receptors.

The mouse model has been widely used as an in vivo mammalian animal model to evaluate the virulence of AIVs. In this study, we used C57BL/6 mice as a mammalian model to study viral pathogenicity. According to the binding affinity and replication kinetics of the viruses investigated in this study, we chose a representative for each of the following AIV H5N1 clades: LP-2006 clade 2.2.1, LP-Q1995D clade 2.2.1.1, LP-S10739C clade 2.2.1.2, and human LP-7271 clade 2.2.1.2. Our results showed that the LP-S10739C causes the highest morbidity and mortality rates as compared with human LP-7271, followed by the LP-2006 clade 2.2.1. This finding is consistent with our further analysis which showed that the amplitude ratios of HPAI H5N1 strains of 2006 clade 2.2.1 and early 2014 clade 2.2.1.2 were comparable in values and higher than 2010 clade 2.2.1.1 strains, but still lower than those of 2015 clade 2.2.1.2 (Figure 6). 

Even though receptor-binding affinities of human LP-7271 and avian LP-S10739C to both human- and avian-type receptors were comparable, the improved replication ability of avian LP-S10739C in mammalian cell lines could augment its pathogenicity in mice and, consequently, provide the possibility for faster adaptation, which might ultimately lead to stronger pathogenicity.

The amplitude ratio for frequencies F (0.236) and F (0.076), which is a marker for the human propensity is 1.34 for S10739C and 1.22 for MOH-7271. Following the ISM sequence analysis of LP-S10739C and human LP-7271, it shows that the key mutations which significantly increase the amplitude ratio, AF (0.236)/AF (0.076), in the HA of H5N1 viruses from 2015 are C4G and L296F. In accordance with our previously reported results [10,13], it can be concluded that these two mutations, which are very rare, could increase the human propensity of this virus. Mutation L296F is present in 12, and C4G in six, of the 7568 H5N1 virus aa sequences in the GISAID depository, respectively. Of note is that five of six C4G mutations were isolated in Egypt. Mutation L296F is present in only one of 1156 viruses from Egypt (data not shown). According to the ISM criteria, these two mutations (single or in combination) will significantly increase its pandemic potential for any H5N1 AIV. Concerning the other two mutations, G269E did not change the amplitude ratio, AF (0.236)/AF (0.076), and K309R is present in many viruses. Conclusively, mutations C4G and L296F, single or in combination, might be considered as potential pandemic markers of H5N1 viruses. In addition, the aa substitutions D154N and D43N, which were fixed in strains from 2014 to 2015 increase the affinity to human receptors and possibly enhance airborne transmissibility in mammals [18,19].

Taken together, the function(s) of these novel aa mutations demands further studies to better understand the 2014 to 2015 human outbreak of HPAI H5N1 viruses in Egypt. Furthermore, we assume that the ISM tool might help to predict the emergence of variant strains of high zoonotic risk and could provide support to the human and veterinary health authorities and vaccine manufacturers to duly plan and respond earlier to newly emerging IAVs with pandemic potential.

## 4. Materials and Methods

### 4.1. Cells and Viruses

Madin-Darby Canine Kidney (MDCK-II) cells, MDCK-SIAT1 cells (MDCK cells engineered to overexpress human-type receptor) [15], human lung adenocarcinoma epithelial cells (A549), and human embryonic kidney cells (293T) were maintained in growth medium (GM). The GM was composed of Dulbecco’s Modified Eagle Medium (DMEM) (Gibco, Invitrogen) containing 100 I.U./mL penicillin, 100 μg/mL streptomycin, and 10% fetal bovine serum (FBS). All cell monolayers were incubated at 37 °C in the presence of 5% CO_2_.

A total of 12 Egyptian H5N1 viruses were isolated and propagated in the allantoic fluids of 10-day-old specific-pathogen-free (SPF) embryonated chicken. Inoculated eggs were incubated for 48 h at 37 °C and then chilled at 4 °C for 4 h before harvesting. Harvested virus stocks were stored at −80 °C for further use.

### 4.2. Sequences

All published sequences of H5N1-HPAIV, isolated in Egypt between 2014 and 2015, were collected from the National Center for Biotechnology Information NCBI Influenza Virus Resource (www.ncbi.nlm.nih.gov/genomes/FLU/) [24] and GISAID EpiFlu database (https://www.gisaid.org/) [25] and were subjected to phylogenetic analysis of HA protein.

### 4.3. The Informational Spectrum Method

Complete description and details of the sequence analysis based on ISM have been published elsewhere [11]. According to this approach, sequences, protein, or DNA sequences are transformed into signals by assignment of numerical values of each element as amino acid or nucleotide, respectively. These values match the electron–ion interaction potential [26,27], determining electronic properties of amino acids and nucleotides for their intermolecular interactions [28]. The obtained signal is, then, decomposed in periodical function by Fourier transformation. The result is a series of frequencies and their amplitudes. The obtained frequencies correspond to the distribution of structural motifs with defined physicochemical characteristics responsible for biological function of the amino acid or nucleotide sequence. When comparing proteins, which share the same biological or biochemical function, the technique allows the detection of code and frequency pairs, which are specific for their common biological properties [11]. The method is insensitive to the location of the motifs, and thus does not require.

### 4.4. The ISM-Based Phylogenetic Analysis

The ISM represented a base for development of the novel phylogenetic algorithm, which analyzes the evolution of the proteins biological function [10,29]. The ISM-based phylogenetic tree was briefly generated using the following algorithm:

1. For each protein sequence *X* calculate its informational spectrum *S_X_*:
  1.1. Transformation of sequence into signal by coding of each amino acid with corresponding EIIP value;
  1.2. Decrease of signal to zero mean;
  1.3. Zero-padding of signal to the length of the longest signal, to set the same resolution to all spectra;
  1.4. Generate the energy density spectrum by applying the fast Fourier transformation (FFT) to the signal.
2. Calculate the distance matrix with the following distance measure between two protein sequences *X* and *Y*:
  (1) $$d\left(X,\;Y\right)=\frac{1}{N}\overset{N/2}{\underset{n=1}{\sum }}\left|S_{X}\left(n\right)-S_{Y}\left(n\right)\right|$$
  where S_*X*_ and S_*Y*_ are the corresponding informational spectra and *N* is the length of the longest sequence.
3. *S_X_* and construct the tree using the unweighted pair group method with arithmetic mean (UPGMA) clustering method.

### 4.5. Generation of H5N1 Viruses Expressing an HA with a Monobasic Cleavage Site

The viral RNA of 12 H5N1 isolates was isolated using the RNeasy kit (Qiagen). To clone the H5-HA gene encoding a monobasic cleavage site, the cDNA was firstly generated from each extracted viral RNA using the uni-12 primer [30]. To specifically amplify the HA segment with a monobasic, site-specific primers were used to convert a multibasic cleavage site to a monobasic cleavage site. Therefore, the HA1 and HA2 coding sequences were amplified separately from the synthesized cDNA, excluding the nucleotide sequence encoding the multibasic cleavage site (PQGEKRRKKR/GLF) for 2.2.1.2 clade [31]. Briefly, the synthesized cDNA (2 μL) were mixed with 25 μL 2x Phusion Master Mix (Thermo Scientific, Waltham, MA, USA) and 3 μL of each primer (40 *p*moles each) [31]. Using nuclease-free water, the total volume was then adjusted to 50 μL. The PCR reactions were kept at 98 °C for 30 sec for initial pre-denaturation, followed by 40 amplification cycles at 98 °C/30 sec, 58 °C/30 sec and 72 °C/3 min. The PCR reactions were then held at 72 °C/10 min for the final extension. The PCR amplicons were eventually purified using the Qiagen Gel Extraction Kit and digested with the FastDigest Esp3I enzyme (Thermo Fisher Scientific, Waltham, MA, USA) according to manufacturer instructions. The digested and purified PCR products were ligated to linearized pMP*ccd*B DNA using T4 DNA ligase, as previously described [32], and transformed into E. coli DH5α.

### 4.6. Generation of Recombinant IAVs (rg-IAVs) and Determination of Replication Kinetics

To generate rg-IAVs, 1 μg of each plasmid DNA, encoding individually the eight viral segments (7 PR8+1 HA), were transfected into a 293T/MDCK-II cell co-culture (ratio 3:1) in 6-well plates, as previously described [32,33,34]. Briefly, a transfection mixture including 180 μL Opti-MEM (Gibco, Invitrogen, Waltham, MA, USA), 8 μg of plasmid DNA mix, and 18 μL Trans-IT kit (Mirus-Bio, Madison, WI, USA) was incubated for 45 min at room temperature (RT). The transfection mixture was then diluted into 1 mL with Opti-MEM medium and transferred to the cells to allow transfection. After 8 h of incubation at 37 °C, the transfection medium was exchanged with 1 mL of infection medium consisting of Opti-MEM medium supplemented with 100 I.U./mL penicillin, 100 μg/mL streptomycin, and 0.2% BSA. The transfected cell co-cultures were further incubated in a humidified CO_2_ incubator at 37 °C. After 12 h of the addition of the infection medium, an additional 1 mL of infection medium containing 2 μg/mL TPCK-treated trypsin was added (Sigma-Aldrich, St Louis, MO, USA). The cell culture supernatant was eventually harvested 48 h after addition of the TPCK-treated trypsin. To reproduce the generated rg-IAV, an aliquot of 1 mL of each supernatant was inoculated into fresh MDCK-II cells. The inoculated cell cultures were then incubated in a humidified CO_2_ incubator for 72 h in the presence of TPCK-treated trypsin (1 μg/mL). The generated recombinant viruses were titrated using TCID_50_ in MDCK-II cells. 

To assess multi-step growth kinetics, confluent monolayers of A549 or MDCK-SIAT1 cells were infected in triplicate with one of the 12 rg viruses at MOI of 0.001. After 1 h of incubation at RT, the inoculum was substituted with infection DMEM medium with 100 I.U./mL penicillin, 100 μg/mL streptomycin, 0.2% BSA, and 1 μg/mL TPCK-treated trypsin. The cells were incubated at 37 °C and the cell culture supernatants were collected at 6, 12, 24, and 36 h post-infection (p.i.). The collected supernatants were then stored at −80 °C. The viral loads in cell culture supernatants were determined by TCID_50_ [35].

### 4.7. Receptor Specificity Assay

Virus receptor specificity was determined as previously described [36]. Ninety six-well fetuin-coated (10 µg/mL) plates were incubated overnight at 4 °C, plates were then blocked with 100 µL phosphate-buffered saline, pH 7.2 (1X PBS) containing 1% BSA for 1 hour at 37 °C. Plates were then washed with ice-cold washing buffer (0.001% Tween 80 in 0.23X PBS) 4 times and incubated overnight with 50 µL of 1X PBS containing 32 hemagglutination units of influenza virus at 4 °C and then washed four times with washing buffer. Biotinylated sialylglycopolymers 3′-sialyllactose (α2,3-SL, Neu5Acα2-3Galβ1-4Glc) or 6′-sialyllactose (α2,6-SL, Neu5Acα2-6Galβ1-4Glc) (Glycotech) were serially diluted in reaction buffer (0.002% Tween 80, 0.02% BSA, 1 µM sialidase inhibitor (zanamivir) and 1X PBS) and were further incubated at 4 °C for 2 h. The plates were washed four times and incubated with 50 µL of reaction buffer containing HRP-conjugated streptavidin (1:2000) at 4 °C for 1 h. Following the final wash, 50 µL of the o-phenylenediamine (OPD) substrate in substrate buffer, composed of phosphate-citrate buffer with sodium perborate capsule (Sigma-Aldrich, Germany) dissolved in ddH_2_O according to manufacturer instructions, was added and incubated for 10 min at RT in dark. The reaction was stopped with 50 µL of 0.5 M sulfuric acid, and absorbance was measured at 490 nm (Biochrom Anthos Zenyth 200rt, USA).

### 4.8. Pathogenicity in Mice

To determine weight and survival in vivo, female C57BL/6 mice (6-to-8-week-old) were divided into 5 groups (5 mice/ group). Four groups of mice were anesthetized with isoflurane and intranasally inoculated with a challenge dose of 30 µL of 10^4.25^/100 µL TCID_50_ from each virus, and an uninfected group was anesthetized and intranasally inoculated with 30 µL of 1X PBS as a control. Upon virus challenge, mice were monitored for 14 days post-infection (dpi) for disease symptoms, weight loss, and mortality. Mortality was recorded either as an actual death or loss of ≥25% body weight, which is the threshold in which animals must be humanely euthanized.

To assess virus replication, groups of three mice were anesthetized with isoflurane and inoculated intranasally with 10^4.25^/100 µL TCID_50_ from each virus in a volume of 30 μL, and were then euthanized on day 3 p.i. Lungs were collected, homogenized in sterile 1X PBS with a Qiagen Tissue Lyser II (Qiagen, Gaithersburg, MD, USA), centrifuged at 2000× *g* for 10 min, and the supernatants were transferred to clean tubes. Virus titers were determined by TCID_50_ assay.

### 4.9. Ethics Statement and Biosafety

The procedures on experimental animals, in our study, were conducted in accordance with the guidelines and regulations of the Egyptian animal welfare regulations and legislation. The animal trial in mice was approved by the Ethics Committee of the National Research Centre (NRC), Egypt (approval number: 16-247). For better biosafety, all selected viruses were genetically manipulated to convert the multibasic cleavage site of the HA of the HPAIV form into a monobasic cleavage site LPAIV form resulting in low pathogenic (LP) rg viruses. All experiments involving low LPAIV were performed in biosafety level 2 containment cabinets.

## Figures and Tables

**Table 1:** IAVs clades and titers in supernatants of MDCK-II-infected cells following 293T/MDCK-II transfection.

rg-H5N1 Strains	Clade	7+1 LP (TCID_50_/100 µL)
A/ chicken/ Egypt/1/2006	2.2.1	10^5.8^
A/chicken/ Egypt/Q1995D/2010	2.2.1.1	10^4.8^
A/duck/Egypt/M2583A/2010	2.2.1.1	10^6.4^
A/chicken/Egypt/M7217B/2013	2.2.1.2	10^5.8^
A/chicken/Egypt/A10540A/2014	2.2.1.2	10^5.3^
A/chicken/Egypt/D10548D/2014	2.2.1.2	10^5.12^
A/chicken/Egypt/D10551C/2014	2.2.1.2	10^4.3^
A/chicken/Egypt/D10552B/2015	2.2.1.2	10^2.42^
A/chicken/Egypt/S10738B/2015	2.2.1.2	10^5.2^
A/chicken/Egypt/S10739C/2015	2.2.1.2	10^5.3^
A/chicken/Egypt/Q10920C/2015	2.2.1.2	10^4.8^
A/duck/Egypt/4/2015	2.2.1.2	10^5.12^
A/ Egypt/MOH-NRC7271/2014	2.2.1.2	10^4.9^

**Table 2:** Amino acid variations among selected LP-H5N1 2014 to 2015 AIVs.

H5N1 Strain	Clade	Amino Acid (aa) Variations *															
		4	12	43	94	120	129	151	154	155	189	193	296	322	373	507	537
2006	2.2.1	C	S	D	N	S	S	I	D	N	R	N	L	Q	K	V	F
Q1995D/2010	2.2.1.1	C	S	D	N	S	S	I	N	N	R	N	L	Q	K	V	F
M2583A/2010	2.2.1.1	C	S	N	N	D	-	T	N	D	R	N	L	Q	K	I	S
M7217B/2013	2.2.1.2	C	S	N	N	D	-	T	S	D	R	N	L	Q	K	I	F
A10540A/2014	2.2.1.2	C	S	N	N	D	-	T	N	D	M	S	L	Q	R	I	S
D10551C/2014	2.2.1.2	C	S	N	N	D	-	T	N	G	R	N	L	K	R	I	S
D10552B/2015	2.2.1.2	G	W	N	N	D	-	T	N	G	R	N	L	K	R	I	S
S10738B/2015	2.2.1.2	C	S	N	N	D	-	T	N	D	R	N	L	Q	K	I	F
S10739C/2015	2.2.1.2	G	S	N	N	D	-	T	N	D	R	N	F	Q	K	I	F
Q10920C/2015	2.2.1.2	C	S	N	T	D	-	T	N	D	R	N	L	Q	R	I	S
A/duck/Egypt/4/2015	2.2.1.2	C	S	N	N	D	-	T	N	D	R	N	L	Q	R	I	S
Human LP-7271/2014	2.2.1.2	C	S	N	N	D	-	T	N	D	R	N	L	Q	R	I	S

* Grey color refers to distinct amino acid changes compared to human LP-7271/2014.
